# Contraceptive ever-use among women of reproductive age in Northern Ghana: a comparative analysis of logistic regression, LASSO penalised regression, and random forest machine learning approaches

**DOI:** 10.3389/fgwh.2026.1857008

**Published:** 2026-09-09

**Authors:** Joseph Lasong, Mavis Kala, Torjim Salifu, Yula Salifu

**Affiliations:** 1 Department of Population and Reproductive Health, School of Public Health, University for Development Studies, Tamale, Ghana; 2 Department of Population Family and Reproductive Health, School of Public Health, University of Ghana, Legon, Ghana

**Keywords:** contraceptive use, family planning, LASSO, machine learning, Northern Ghana, random forest, women of reproductive age

## Abstract

**Background:**

Contraceptive uptake in Northern Ghana remains low despite high awareness, suggesting barriers not fully captured by additive models. This study compared logistic regression, LASSO, and Random Forest to predict contraceptive ever-use and identify determinants among women in Tamale Metropolis.

**Methods:**

A facility-based cross-sectional survey (January–April 2023) included 414 women aged 15–49 from three public health facilities. Outcome: contraceptive ever-use. Twelve predictors included knowledge/attitude scores, wealth tertile, education, parity, marital status, occupation, age, cultural barriers, health worker attitudes, and information source. Model discrimination was assessed via AUC with 95% CIs; LASSO used 10-fold cross-validation (*λ*.min=0.012); Random Forest used 500 trees.

**Results:**

Random Forest achieved highest discrimination (AUC=0.865, 95% CI:0.83–0.90), outperforming logistic regression (AUC=0.727) and LASSO (AUC=0.723) (both *p* < 0.001); logistic and LASSO did not differ (*p* = 0.91). Logistic regression identified five independent predictors: positive attitudes (AOR=1.53), education above primary (AOR=2.72), parity (AOR=2.95), health worker as information source (AOR=2.07), and richest wealth tertile inversely associated (AOR=0.49). Knowledge was non-significant in regression but ranked second in Random Forest importance, suggesting non-linear/interaction effects. LASSO retained nine predictors, dropping cultural barriers, health worker attitudes, and age.

**Conclusions:**

Random Forest provided better discrimination and may complement regression for prediction, though regression remains essential for interpretable effect estimates. Attitude change, education, and counselling quality are priority targets. The inverse wealth association warrants qualitative investigation, as polygyny, religiosity, and partner dynamics were unmeasured.

## Background

Universal access to modern contraception is a foundational component of Sustainable Development Goal (SDG) 3 (good health and well-being) and SDG 5 (gender equality and women's empowerment), and is central to reducing preventable maternal mortality, enabling women's economic participation, and preventing unintended pregnancies (1, 2). The United Nations Population Fund (UNFPA) estimates that meeting all unmet need for family planning in low- and middle-income countries would avert approximately 67 million unintended pregnancies and 67,000 maternal deaths annually (3). Despite decades of programmatic investment, sub-Saharan Africa continues to record the lowest modern contraceptive prevalence rate (mCPR) globally, approximately 25% among married women, alongside the highest total fertility rate (4.4 births per woman), reflecting persistent structural, attitudinal, and health-system barriers to uptake (4, 5).

Ghana's national mCPR has improved substantially, rising from 18% in 1993 to 47% in 2022 (6). This aggregate progress, however, masks marked sub-national disparities. The Northern, Savanna, and North East regions, collectively referred to here as Northern Ghana, record among the lowest mCPRs in the country (*N*orthern Region: 17%; North East Region: 15%) and the highest fertility rates (5.6–6.6 births per woman, approximately 1.1 times the national average), reflecting a persistent reproductive health equity gap (6, 7). The Tamale Metropolis Annual Performance Health Reviews further documented a consistent year-on-year decline in contraceptive acceptability between 2019 and 2021 (39% → 25% → 23.6%), indicating deteriorating service uptake despite high awareness (8).

A companion study using the same dataset as the present investigation, Kala et al. (7), published in *Contraception and Reproductive Medicine*, reported that among 421 women attending primary care facilities in the Tamale Metropolis, contraceptive acceptability was 75.3% while current utilisation was only 40.1%, an intention–behaviour gap of 35 percentage points. Determinants of current utilisation in that study included age, marital status, household wealth, paternal education, and health worker attitudes, while acceptability was predicted by occupation, knowledge, health worker information access, and polygyny. That analysis relied on conventional multivariable logistic regression, which assumes linearity on the log-odds scale, is susceptible to overfitting as the number of predictors grows and does not perform automatic variable selection. It is therefore possible that non-linear and interaction-mediated predictor effects, including knowledge–attitude interactions and wealth-stratified patterns, were not fully captured.

Machine learning approaches offer distinct methodological advantages for analysing complex health behaviour data. LASSO penalised regression (9) performs simultaneous variable selection and regularisation, shrinking the coefficients of less important variables toward, and in some cases exactly to, zero. Random Forest (10), an ensemble method based on bootstrap aggregation of decision trees, captures non-linear relationships and higher-order interactions without requiring a pre-specified functional form, and yields a variable importance ranking that does not depend on parametric assumptions. Both methods have been applied to reproductive health prediction in high-income settings (11, 12) but remain underutilised in sub-Saharan African reproductive health research, where their use could strengthen both analytical rigour and the practical targeting of interventions.

The present study addresses this gap by applying and comparing logistic regression, LASSO, and Random Forest to predict contraceptive ever-use – defined as lifetime exposure to any modern or traditional contraceptive method – among 414 women of reproductive age in the Tamale Metropolis. Ever-use, rather than current use or acceptability, was selected as the outcome to capture cumulative lifetime contraceptive exposure and to identify factors shaping the initial adoption decision. The specific objectives were to: (1) describe sample characteristics and the prevalence of contraceptive ever-use; (2) identify independent predictors of ever-use using logistic regression and LASSO; (3) compare model discrimination across the three approaches using the AUC; and (4) assess whether Random Forest reveals predictor importance patterns not detected by conventional regression.

## Conceptual framework

This study applies the Information–Motivation–Behavioural Skills (IMB) model (13) as its theoretical framework. The IMB model proposes that health behaviour change, including contraceptive uptake, is determined primarily by three constructs: (1) information (factual knowledge about the behaviour and its consequences); (2) motivation (personal attitudes and perceived social norms); and (3) behavioural skills (the practical capacity and self-efficacy required to perform the behaviour). The model predicts that information and motivation influence behaviour largely through their effects on behavioural skills, and that motivation becomes the stronger predictor when the target behaviour is complex or socially contested. This is a condition that characterises contraceptive use in the conservative Islamic communities that predominate in the Tamale Metropolis. The IMB model has been validated in contraceptive behaviour research across sub-Saharan African settings (14, 15) and provides the rationale for treating knowledge and attitude as primary predictors, with structural access variables specified as more distal enabling factors.

## Methods

### Study design, setting, and population

This cross-sectional study was conducted between January and April 2023 in the Tamale Metropolis, Northern Region, Ghana. The Tamale Metropolis, one of 16 administrative regional capitals, covers 646.9 km^2^ (9°16′–9°34′ N latitude; 0°36′–0°57′ W longitude) and has an estimated population of 374,744 [male: 185,051 [49.4%]; female: 189,693 [50.6%]], served by eight public health facilities, more than twenty private facilities, and one facility operated by the Christian Health Association of Ghana (7). Participants were women of reproductive age (15–49 years) attending three primary public health facilities including Nyohini, Bilpiela, and Vitting Clinics, randomly selected from eligible community facilities within the Metropolis. Full detail on the data collection instruments, sampling procedures, and quality assurance protocols for this dataset is provided in the companion publication (7), from which the present study draws secondary data for a distinct, machine-learning-focused analytical objective.

### Eligibility and sample size

Inclusion criteria were female sex; age 15–49 years; actively seeking care at one of the three selected facilities; residence in the Metropolis for ≥6 months; and provision of written informed consent (with guardian consent plus participant assent for those aged 15–17). Exclusion criteria were history of hysterectomy and unwillingness to participate. Sample size was estimated using Yamane's formula (*n* = N/[1 + N(e)^2^] (16);), where *N* = 10,815 (the estimated population of reproductive-aged women in the three catchment communities) and e = 0.05, yielding a minimum of 386, inflated by 10% for non-response to 424 (7). This calculation was designed to yield a precise prevalence estimate for the parent study and was not formally powered for multivariable predictive modelling; this is acknowledged as a limitation of the present secondary analysis. Of 424 women targeted, 421 were enrolled (99.3% response rate), and 414 remained after excluding three respondents with more than 20% missing data. For the machine learning analysis, 387 complete cases were available after listwise deletion of records with any missing covariate value (93.5% retention from 414); further detail is available in Kala et al. (7).

### Participant sampling

Participants were distributed across the three facilities using quota sampling, with quotas allocated in proportion to the population of reproductive-aged women served by each facility {facility X quota=[X/(X + Y + Z)] × total sample size}. Within each facility, a systematic random selection procedure was used: a random starting point was chosen from among the first three eligible women, and every third subsequent woman was recruited until the facility quota was reached; where a selected woman declined or did not meet the inclusion criteria, the next eligible woman was approached instead. A full description of the sampling procedure is provided in Kala et al. (7).

### Data collection

Six enumerators holding Master of Public Health degrees, fluent in English and Dagbani, were trained for two days prior to data collection. The structured questionnaire was administered via an electronic KoboCollect form: literate participants self-administered the survey through a shared digital link, while enumerators orally administered and read back all responses for non-literate participants. Interviews lasted 15–30 min. The instrument was pretested among 30 women in Sagnarigu, a community with similar characteristics to the study area (pilot data were excluded from the main analysis) and was back translated between Dagbani and English to verify accuracy. Reporting followed the Strengthening the Reporting of Observational Studies in Epidemiology (STROBE) guidelines (17).

### Outcome Variable

Contraceptive ever-use was assessed with the question, ‘Have you ever used any form of contraception in your life?’, dichotomised as 1 (yes) or 0 (no). Prevalence was 56.3% (*n* = 233) in the full sample (*N* = 414) and 56.6% (*n* = 219) in the analytical sample (*n* = 387). Ever-use captures lifetime initiation of any modern method [implant, injectable, pill, intrauterine device (IUD), condom] or traditional method (abstinence, lactational amenorrhoea), providing a broader indicator of lifetime engagement with family planning than current use and enabling analysis of the factors shaping initial adoption (7, 18).

### Predictor variables

#### Contraceptive knowledge score

A six-item score assessed factual knowledge of long-acting reversible contraceptive methods (LARCMs): (1) correct identification of LARCMs (implant or IUD) vs. non-LARCMs (injectables, pills, or other methods); (2) belief that modern methods are safer than combined oral contraceptives; (3) knowledge that LARCM effects are reversible; (4) understanding that LARCMs do not prevent sexually transmitted infections (STIs); (5) correct identification of the uterus as the IUD insertion site; and (6) knowledge that modern contraception does not confer 100% protection. Each item was scored 1 (correct) or 0 (incorrect/don't know) and summed into a composite score of 0–6 (observed range: 1–6). Cronbach's alpha for the composite was 0.31. This low value is expected for a factual knowledge measure whose items span distinct content domains (method identification, safety, mechanism, effectiveness) rather than a single underlying latent trait and is consistent with recommendations that such formative indices, where items are causes rather than reflections of a common construct – need not, and often should not, exhibit high internal consistency (19). The implications of this limitation for interpreting the knowledge-score findings are considered further in the Discussion and Limitations.

#### Attitude score

Six negative attitude statements toward family planning were assessed: that it (1) predisposes to infertility; (2) affects one's relationship with husband and his family; (3) causes weight gain; (4) causes menstrual irregularity; (5) is not acceptable by one's religion or culture; and (6) makes people promiscuous. Items were reverse-coded (endorsement of a negative belief=0; rejection=1) and summed into a score of 0–6, with higher values indicating more positive attitudes. The most prevalent negative attitudes were beliefs about menstrual irregularity (87.2%), weight gain (79.7%), and promiscuity (67.9%), indicating a substantial burden of deterrent attitudes in this population.

#### Household wealth tertile

Household wealth was assessed following Demographic and Health Survey (DHS) methodology (20). Principal components analysis (PCA) was conducted on nine household asset variables, selected after excluding near-constant items (ownership >90% or <10%): colour television, sewing machine, mattress, bed, table, refrigerator, bicycle, car/truck, and livestock. Mode imputation was applied for variables with minimal missing data. The first principal component served as the wealth index, and participants were classified into tertiles: poor (*n* = 141), medium (*n* = 150), and rich (*n* = 96 in the analytical sample).

#### Sociodemographic and health system variables

Age group was categorised as <20, 20–30, or 30–40 years. Marital status was dichotomised as currently married (including cohabiting) vs. not currently married (single, divorced, widowed). Education was dichotomised as formal education above primary level (Junior High School, Senior High School/technical/vocational, or tertiary=1) vs. no formal education or primary only (= 0), reflecting a threshold considered meaningful for literacy and health-seeking capacity. Occupational status was coded as currently employed in any sector (= 1) vs. unemployed (= 0). Parity was coded as having at least one living child (= 1) vs. nulliparous (= 0). Cultural barriers, health worker attitude barriers, and health worker as the primary information source were each coded as binary indicators, described in Table 1.

**Table 1:** Sociodemographic, reproductive, knowledge, attitudinal, and health system characteristics of study participants (*N* = 414).

Variable	*n*	%
Age group (years)		
<20	29	7.0
20–30	233	56.3
30–40	151	36.5
Religion		
Christianity	54	13.0
Islam	360	87.0
Marital status		
Currently married	376	90.8
Single	33	8.0
Divorced/widowed	5	1.2
Educational attainment		
No formal education	51	12.3
Primary only	65	15.7
Junior High School	76	18.4
Secondary/Technical/Vocational	107	25.9
Tertiary	115	27.8
Occupational status		
Civil servant	113	27.5
Trader	79	19.2
Farmer	48	11.7
Unemployed	32	7.8
Seamstress/Other	139	33.9
Household wealth tertile (PCA-derived)		
Poor	141	34.1
Medium	137	33.1
Rich	136	32.9
Area of residence		
Urban	342	83.2
Rural	35	8.5
Peri-urban	34	8.3
Knowledge items (correct response)		
Aware of contraception	404	97.6
Knows reversible LARC methods (>3 yrs)	378	91.3
LARCMs safer than COCs	331	80.0
Contraceptive effects reversible	334	81.5
LARCMs do not prevent STIs	373	90.5
IUD insertion site=uterus	270	65.4
Modern contraception not 100% effective	311	75.1
Negative attitude items (endorsed=barrier)		
FP predisposes to infertility	130	31.4
FP affects relationship with husband	161	39.1
FP causes weight gain	329	79.5
FP causes menstrual irregularity	360	87.2
FP not acceptable by religion/culture	108	26.3
FP makes people promiscuous	281	67.9
Contraceptive behaviour and health system factors		
Ever used any contraception	233	56.3
Currently on any contraceptive	59	14.3
Willing to start contraception now	275	66.9
Cultural beliefs prevent FP use	107	25.9
HW attitudes prevent FP use	72	17.4
Health facility in area providing FP	392	94.7
FP services affordable	356	86.0
Health worker=primary info source	334	81.1
Contraceptive methods ever used (among ever-users; *n* = 233)		
Injectables	177	45.3
Condom	98	25.1
Implant	59	15.1
Oral pill	44	11.3
Abstinence	13	3.3

FP, family planning; HW, health worker; LARC, long-acting reversible contraceptive; LARCM, long-acting reversible contraceptive method; STI, sexually transmitted infection; COC, combined oral contraceptive; PCA, principal components analysis. Proportions for knowledge items reflect correct/appropriate responses; negative attitude items reflect endorsed beliefs that constitute barriers to uptake.

### Statistical analysis

#### Descriptive and bivariate statistics

Frequencies and proportions were computed for categorical variables and means and standard deviations for continuous variables. Contraceptive ever-use prevalence was computed for the full sample (*N* = 414) and the analytical sample (*n* = 387). Bivariate associations between each predictor and ever-use were examined using Pearson chi-square tests (categorical predictors) and independent-samples t-tests (continuous predictors), with statistical significance set at *p* < 0.05.

#### Missing data

Of the 414 participants retained after initial data cleaning, 27 (6.5%) had at least one missing value on the twelve candidate predictors and were excluded from the multivariable analyses by listwise deletion, yielding the analytical sample of 387. This approach assumes that data are missing completely at random. A formal comparison of sociodemographic characteristics between included and excluded participants was not performed in this analysis and is noted as a limitation, since undetected systematic missingness could bias the reported associations.

#### Model 1: multivariable logistic regression

A multivariable logistic regression model was fitted with all twelve predictors entered simultaneously (enter method), estimating adjusted associations while controlling for all other covariates without an automated selection algorithm that could introduce instability (21). Wealth tertile was entered as two dummy variables (medium and rich, with poor as reference). Results are presented as adjusted odds ratios (AOR) with 95% profile likelihood confidence intervals and two-sided *p*-values.

#### Model 2: LASSO penalised logistic regression

LASSO logistic regression (9) was fitted using the glmnet package (22) in R. LASSO adds an L1 penalty (*λ* × *Σ*|*β*ⱼ|) to the log-likelihood, performing simultaneous variable selection (shrinking some coefficients exactly to zero) and regularisation (penalising coefficient magnitude to reduce overfitting). The optimal penalty parameter was selected by 10-fold cross-validation using the minimum cross-validated deviance criterion (*λ*.min=0.012). Variables with non-zero coefficients at *λ*.min were considered retained. LASSO coefficients represent penalised log-odds rather than directly interpretable odds ratios, but their rank order by absolute magnitude provides a valid importance ranking.

#### Model 3: random forest classifier

A Random Forest classifier (10) was fitted using the randomForest package (23) with 500 classification trees and the default number of candidate predictors per split (√p). Random Forest is a bootstrap-aggregating ensemble that builds multiple decision trees on bootstrapped data subsets and uses out-of-bag samples to estimate prediction error, capturing non-linear relationships and interactions without a pre-specified functional form. Variable importance was quantified as the mean decrease in Gini impurity – the total reduction in node impurity attributable to each variable, averaged across all 500 trees. As a predictive contribution metric rather than a confounder-adjusted effect estimate, Gini importance indicates that a variable assists classification accuracy but does not, by itself, establish the direction, linearity, or causal structure of that contribution.

#### Model validation and comparison

Discrimination was quantified for each model using the AUC, computed from model-predicted probabilities for logistic regression and LASSO, and from out-of-bag class probability estimates for Random Forest; the latter provides an internally cross-validated performance estimate without requiring a separate holdout partition. Ninety-five percent confidence intervals for each AUC were calculated using the Hanley–McNeil method (24), based on the number of ever-users (*n* = 219) and never-users (*n* = 168) in the analytical sample. Pairwise differences between AUCs were evaluated with a two-sample z-test that treats the compared AUCs as statistically independent; because all three models were fitted to the same sample, this test is conservative, since the true positive correlation between model predictions would be expected to reduce the standard error of the difference. Calibration, which is the agreement between predicted probabilities and observed outcome frequencies, was not formally assessed in this analysis and is noted as a limitation, particularly relevant should these models be developed further for programme-level risk targeting.

#### Model comparison and software

AUC values were interpreted using conventional benchmarks: 0.70–0.79 = acceptable; 0.80–0.89 = good; 0.90–1.00 = excellent (25). The random seed was set to 42 for reproducibility. All analyses were conducted in R version 4.5.2 (26).

#### Ethical considerations

The study received approval from the Institutional Ethics Review Board of the University for Development Studies (UDS/RB/018/24). Administrative permission was obtained from the heads of all participating facilities. Written informed consent was obtained from all participants ≥18 years; written guardian consent plus participant assent was obtained for participants aged 15–17. Participants were informed of their right to withdraw at any time without consequence, and all data were anonymised and stored securely. The study complied with the Declaration of Helsinki (2013 amendment) and the STROBE reporting guidelines (17).

## Results

### Sample characteristics

Table 1 presents the sociodemographic, knowledge, attitudinal, and health system characteristics of the 414 enrolled participants. Most participants were aged 20–30 years (56.3%), followed by 30–40 years (36.5%) and under 20 years (7.0%). Religion was predominantly Islam (87.0%), consistent with the demographic profile of the Tamale Metropolis. Most participants were currently married (90.8%), with 8.0% single and 1.2% divorced or widowed. Formal education above primary level was reported by 71.98% of participants (Junior High School: 18.4%; Secondary/Technical/Vocational: 25.9%; Tertiary: 27.8%); 12.3% had no formal education and 15.7% had primary schooling only. The sample was predominantly urban (83.2%), with 8.5% rural and 8.3% peri urban. Civil servants (27.5%) and traders (19.2%) were the largest occupational categories. Among the 393 participants with non-missing parity data, 90.1% reported at least one living child. Injectables were the most cited method ever used (45.3%), followed by condoms (25.1%), implants (15.1%), and pills (11.3%). Health workers were the dominant source of LARCM information (81.1%).

### Model 1: multivariable logistic regression results

Table 2 presents the fully adjusted logistic regression results. Five predictors were statistically significant (*p* < 0.05). Positive attitude toward contraception was the strongest positive predictor (AOR=1.53, 95% CI: 1.29–1.83, *p* < 0.001): each one-unit increase in attitude score was associated with 53% greater odds of ever-use, adjusting for all other variables. Formal education above primary level was associated with 2.72-fold greater odds (95% CI: 1.65–4.54, *p* < 0.001). Having at least one child was associated with nearly three-fold greater odds of ever-use (AOR=2.95, 95% CI: 1.20–7.56, *p* = 0.020). Receipt of family planning information from a health worker was associated with roughly twice the odds of ever-use (AOR=2.07, 95% CI: 1.11–3.91, *p* = 0.022). Membership in the richest wealth tertile was associated with significantly lower odds of ever-use relative to the poorest tertile (AOR=0.49, 95% CI: 0.27–0.89, *p* = 0.019). Given the modest subgroup size (*n* = 96), this estimate should be interpreted alongside its comparatively wide confidence interval. Knowledge score was not independently significant (AOR=1.05, 95% CI: 0.85–1.30, *p* = 0.642), nor were age group, marital status, occupational status, cultural barriers, or health worker attitude problems. The logistic regression model achieved an AUC of 0.727 (95% CI: 0.68–0.78).

**Table 2:** Adjusted logistic regression results: predictors of contraceptive ever-use (analytical sample, *n* = 387).

Predictor	AOR	95% CI	p	Sig.	LASSO *β* (retained)
Knowledge score (per unit)	1.051	0.853–1.296	0.642	No	Yes (0.014)
Attitude score (per unit)	1.532	1.289–1.832	<0.001	Yes	Yes (0.379)
Wealth: medium vs. poor	0.658	0.379–1.138	0.135	No	Yes (−0.196)
Wealth: rich vs. poor	0.494	0.273–0.886	0.019	Yes	Yes (−0.454)
Age group	0.972	0.607–1.558	0.907	No	Eliminated
Currently married	0.509	0.131–1.826	0.309	No	Yes (−0.175)
Education: above primary	2.716	1.645–4.535	<0.001	Yes	Yes (0.876)
Occupational status: employed	1.843	0.731–4.804	0.200	No	Yes (0.411)
Has ≥1 living child	2.950	1.202–7.557	0.020	Yes	Yes (0.741)
Cultural barriers present	0.935	0.551–1.596	0.804	No	Eliminated
HW attitude problem	0.983	0.524–1.857	0.957	No	Eliminated
HW = primary info source	2.074	1.112–3.913	0.022	Yes	Yes (0.610)

AOR, adjusted odds ratio; CI, 95% profile likelihood confidence interval; HW, health worker. Reference categories: wealth, poor tertile; education, no formal or primary only; marital status, not currently married; occupation, unemployed; parity, nulliparous. Model AUC, 0.727 (95% CI: 0.68–0.78). LASSO column reports the penalised coefficient at *λ*.min, 0.012; ‘Eliminated’ denotes a coefficient shrunk to exactly zero.

### Model 2: LASSO Variable selection results

Table 3 presents LASSO results at the optimal penalty (*λ*.min=0.012, selected by 10-fold cross-validation). LASSO retained nine of twelve predictors, eliminating cultural barriers, health worker attitude problems, and age group. Ranked by absolute penalised coefficient, education had the largest coefficient (*β* = 0.876), followed by parity (0.741), health worker information source (0.610), rich wealth tertile (−0.454), occupational status (0.411), attitude score (0.379), medium wealth tertile (−0.196), marital status (−0.175), and knowledge score (0.014, near-zero but retained). All directions of association were consistent with the logistic regression model. The LASSO AUC was 0.723 (95% CI: 0.67–0.77), marginally below logistic regression, consistent with the coefficient shrinkage that trades within-sample discrimination for improved generalisability.

**Table 3:** LASSO-selected predictors of contraceptive ever-use at *λ*.min=0.012 (*n* = 387).

Variable	LASSO β	Direction	LR AOR (p)	Status
Education: above primary	0.876	Positive	2.716 (*p* < 0.001)	Retained; significant in LR
Has ≥1 living child	0.741	Positive	2.950 (*p* = 0.020)	Retained; significant in LR
HW = primary info source	0.610	Positive	2.074 (*p* = 0.022)	Retained; significant in LR
Wealth: rich vs. poor	−0.454	Negative	0.494 (*p* = 0.019)	Retained; significant in LR
Occupational status: employed	0.411	Positive	1.843 (*p* = 0.200)	Retained; ns in LR
Attitude score	0.379	Positive	1.532 (*p* < 0.001)	Retained; significant in LR
Wealth: medium vs. poor	−0.196	Negative	0.658 (*p* = 0.135)	Retained; ns in LR
Currently married	−0.175	Negative	0.509 (*p* = 0.309)	Retained; ns in LR
Knowledge score	0.014	Positive	1.051 (*p* = 0.642)	Retained (near-zero); ns in LR
Age group	0.000	–	0.972 (*p* = 0.907)	Eliminated
Cultural barriers	0.000	–	0.935 (*p* = 0.804)	Eliminated
HW attitude problem	0.000	–	0.983 (*p* = 0.957)	Eliminated

HW, health worker; LR, logistic regression; ns, not significant; AOR, adjusted odds ratio. LASSO coefficients are on the penalised log-odds scale. LASSO model AUC=0.723 (95% CI: 0.67–0.77). LR AORs reproduced from Table 2 for comparison.

### Model 3: random forest Variable importance

Table 4 presents Random Forest variable importance by mean decrease in Gini impurity. Attitude score ranked first (Gini=30.01), followed by knowledge score (24.68), education (9.43), age group (8.96), and health worker information source (8.71). Cultural barriers (7.85), medium wealth tertile (7.18), and rich wealth tertile (6.42) showed intermediate importance. Health worker attitude problems (5.74), parity (4.58), occupational status (3.39), and marital status (2.67) had the lowest importance values. As noted in the Methods, these values quantify predictive contribution and should not be read as confounder-adjusted effect sizes or as evidence of a causal pathway. The Random Forest AUC was 0.865 (95% CI: 0.83–0.90), higher than both logistic regression and LASSO. The most notable discordance between Random Forest and logistic regression concerns knowledge score: non-significant in logistic regression (*p* = 0.642) but ranked second in Random Forest importance. This pattern is consistent with a non-linear or interaction-mediated association that additive models do not capture, although this interpretation cannot be confirmed without directly modelling candidate interaction terms.

**Table 4:** Random forest variable importance: mean decrease in gini impurity (*n* = 387, 500 trees).

Variable	Mean decrease Gini	RF rank	LR AOR (p)	Concordance with LR
Attitude score	30.01	1	1.532 (*p* < 0.001)	Concordant – significant in both
Knowledge score	24.68	2	1.051 (*p* = 0.642)	Discordant – important in RF only
Education: above primary	9.43	3	2.716 (*p* < 0.001)	Concordant – significant in both
Age group	8.96	4	0.972 (*p* = 0.907)	Discordant – important in RF only
HW = primary info source	8.71	5	2.074 (*p* = 0.022)	Concordant – significant in both
Cultural barriers	7.85	6	0.935 (*p* = 0.804)	Partial – moderate in RF only
Wealth: medium vs. poor	7.18	7	0.658 (*p* = 0.135)	Partial – moderate in RF only
Wealth: rich vs. poor	6.42	8	0.494 (*p* = 0.019)	Concordant – significant in both
HW attitude problem	5.74	9	0.983 (*p* = 0.957)	Discordant – modest in RF only
Has ≥1 living child	4.58	10	2.950 (*p* = 0.020)	Concordant – significant in both
Occupational status: employed	3.39	11	1.843 (*p* = 0.200)	Partial – low in RF only
Currently married	2.67	12	0.509 (*p* = 0.309)	Concordant – non-significant in both

HW, health worker; LR, logistic regression; RF, random forest; AOR, adjusted odds ratio. Mean decrease in Gini reflects total reduction in node impurity across all 500 trees attributable to each variable; higher values indicate greater predictive contribution. Random Forest AUC, 0.865 (95% CI: 0.83–0.90).

### Model performance comparison

Table 5 summarises comparative model performance. Random Forest (AUC=0.865, 95% CI: 0.83–0.90) outperformed both logistic regression (AUC=0.727, 95% CI: 0.68–0.78) and LASSO (AUC=0.723, 95% CI: 0.67–0.77) by conventional benchmarks, achieving good-to-excellent discrimination against acceptable discrimination for the two regression-based models. Pairwise z-tests (Hanley–McNeil standard errors, treated conservatively as independent) indicated that the Random Forest advantage was statistically significant relative to both logistic regression (*Δ*AUC = 0.138, z = 4.44, *p* < 0.001) and LASSO (*Δ*AUC = 0.142, z = 4.55, *p* < 0.001), while logistic regression and LASSO did not differ meaningfully from one another (*Δ*AUC = 0.004, z = 0.11, *p* = 0.91), consistent with their shared assumption of log-odds linearity.

**Table 5:** Comparative model performance: area under the receiver operating characteristic curve (AUC).

Model	AUC	95% CI	Interpretation	Key features
Logistic regression	0.727	0.68–0.78	Acceptable	5 significant predictors; parametric; assumes log-odds linearity
LASSO (*λ*.min=0.012)	0.723	0.67–0.77	Acceptable	9 predictors retained; regularised; automatic variable selection
Random Forest (500 trees)	0.865	0.83–0.90	Good–excellent	Highest AUC; non-parametric; captures non-linear effects and interactions

AUC interpretation benchmarks (25): 0.70–0.79 = acceptable; 0.80–0.89 = good; 0.90–1.00 = excellent. 95% CIs computed using the Hanley–McNeil method (24). All models trained and evaluated on the analytical sample (*n* = 387).

## Discussion

This study applied and compared logistic regression, LASSO, and Random Forest to predict contraceptive ever-use among women of reproductive age in Northern Ghana. The three-model design enables direct methodological comparison and generates evidence on predictor pathways, particularly non-linear and interaction-mediated ones, that a single conventional model cannot fully reveal.

Positive attitude toward contraception was the strongest significant predictor in logistic regression (AOR=1.53 per unit, *p* < 0.001) and ranked first in Random Forest importance (Gini=30.01), a convergent result across two methodologically distinct approaches. This is consistent with the IMB model's prediction that motivation is the dominant driver of health behaviour in contexts where the behaviour is socially contested (13). Descriptive findings illustrate the scale of the attitudinal barrier in this population: 87.2% of participants believed family planning causes menstrual irregularity, 79.5% believed it causes weight gain, and 67.9% believed it makes people promiscuous. Ahinkorah (27), in a pooled analysis of 29 sub-Saharan African DHS datasets, similarly identified fear of side effects as among the most cited barriers to contraceptive uptake. Community-based interventions such as peer counselling by satisfied users and structured myth-busting dialogues have shown stronger evidence for shifting contraceptive attitudes than knowledge-only education campaigns (28), suggesting that attitude-focused programming targeting menstrual irregularity and weight-gain myths specifically should be prioritised in family planning communication for the Tamale Metropolis.

Contraceptive knowledge score was non-significant in logistic regression (AOR=1.05, *p* = 0.642) and retained by LASSO only with a near-zero coefficient (*β* = 0.014) yet ranked second in Random Forest importance (Gini=24.68). This pattern has been documented elsewhere: Speiser et al. (11) found that Random Forest routinely identifies variables with non-linear effects as important even when they are non-significant in logistic regression, and Austin and Tu (12) showed that Random Forest outperforms logistic regression specifically when true predictor–outcome relationships are non-linear or interaction-mediated. A plausible, though untested, explanation in the present context is that knowledge matters mainly in combination with other enabling factors. For instance, a woman with high knowledge but negative attitudes or no health worker contact may be no more likely to have ever used contraception than a woman with low knowledge, whereas high knowledge combined with positive attitudes and health worker engagement may substantially raise the probability of use. Such interaction effects would be invisible to an additive logistic model but detectable by Random Forest's recursive partitioning; testing this explanation directly would require fitting logistic regression models with pre-specified knowledge×attitude and knowledge×health-worker-contact interaction terms, which was beyond the scope of the present analysis. This discordance should also be interpreted in light of the knowledge scale's low internal consistency (*α* = 0.31): because the composite score combines conceptually distinct items (method identification, safety perceptions, mechanism knowledge, effectiveness knowledge), a single *p*-value from an additive model may understate its true relevance, while Random Forest's importance ranking, which does not depend on a single linear combination of items, may be more sensitive to the contribution of the score as a whole. This extends the companion analysis by Kala et al. (7), which found knowledge to be a strong determinant of acceptability (AOR=3.67) but did not examine possible interactive effects on utilisation.

Formal education above primary level was independently associated with 2.72-fold greater odds of ever-use (*p* < 0.001), the second-largest positive association in logistic regression, and was retained by LASSO with the largest coefficient of any predictor (*β* = 0.876). This is among the most consistent findings in the global reproductive health literature, replicated across methodologies, populations, and measurement approaches in sub-Saharan Africa (27, 29). Education is understood to operate through several pathways: greater health literacy, stronger negotiating capacity within intimate relationships, more frequent contact with formal health information channels, and higher occupational status supporting independent access to services. The magnitude observed here is at the upper end of published estimates from Northern sub-Saharan African populations, plausibly reflecting the scale of educational disadvantage in this sample: 12.3% of participants had no formal education and a further 15.7% had only primary schooling, placing almost 28% of the sample outside the educational pathway associated with contraceptive initiation. Kala et al. (7) did not find respondent's own education to be a significant predictor of current utilisation, although paternal education was significant in that analysis (AOR=2.01–2.86). Taken together, these results suggest that women's own education may matter more for initial adoption than for continuation, which may depend more heavily on partner-level factors, though this comparison is drawn across two different outcomes in the same sample rather than tested directly.

Having at least one living child was associated with nearly three-fold greater odds of ever-use (AOR=2.95, *p* = 0.020; LASSO *β* = 0.741, the second-largest retained coefficient). This is consistent with two complementary mechanisms documented in the sub-Saharan African literature: prior contact with maternal health services, including post-natal contraceptive counselling, and heightened motivation to regulate subsequent fertility following childbirth (27). A practical implication is that post-partum family planning counselling, delivered through both facility-based post-natal care and community health worker home visits, represents a high-leverage moment for contraceptive initiation. Given that injectables were the most commonly reported method ever used (45.3%), post-partum counselling that also introduces higher-efficacy, longer-acting methods (implants, IUDs) could help address the contraceptive failure documented in the companion study, in which 52.7% of women who conceived their last pregnancy had been using contraception beforehand (7).

Receipt of contraceptive information primarily from a health worker was independently associated with twice the odds of ever-use (AOR=2.07, *p* = 0.022; LASSO *β* = 0.610; Random Forest rank=5th), a result that was consistent across all three models. This aligns with qualitative evidence on the role of health worker engagement in triggering contraceptive initiation in Northern Ghana (30), sub-Saharan Africa more broadly (31, 32), and other settings (33). Health workers were already the dominant information source in this sample (81.1%, compared with 12.6% for friends and 3.4% for television); the persistence of an independent association after adjustment suggests that the substance and quality of counselling, rather than mere contact, may be the operative mechanism, consistent with the IMB model's distinction between information provision and motivational engagement. By contrast, health worker attitude problems were not significant in any model and were eliminated by LASSO, diverging from the Kala et al. (7) finding that health worker attitudes were a significant barrier to current utilisation (AOR=0.23, *p* < 0.001). One possible explanation is that provider attitude barriers matter more for sustaining use than for initiating it, though this distinction was not tested directly and should be treated as a hypothesis.

The richest household wealth tertile was associated with significantly lower odds of ever-use relative to the poorest tertile (AOR=0.49, *p* = 0.019), and LASSO retained rich wealth with the fourth-largest absolute coefficient (*β* = −0.454). This directly contrasts with Kala et al. (7), which found both medium (AOR=1.93) and rich (AOR=2.19) wealth tertiles positively associated with current utilisation in the same population. The explanations offered below are speculative and intended to generate hypotheses for future qualitative research rather than to establish a confirmed mechanism, since polygyny, religious practice intensity, and partner-level dynamics were not directly measured in this study. One possibility is that richer households in Northern Ghana are more frequently polygynous. Polygyny was negatively associated with acceptability in the companion analysis [AOR=0.61 (7);], and that women in polygynous unions may experience reduced reproductive autonomy or greater pressure to demonstrate fertility to secure their position within the household. A second possibility is that wealthier women in this predominantly Islamic population are more embedded in conservative religious networks that discourage contraceptive use (34). If richer women who do initiate contraception are nonetheless better resourced to sustain and access services once they do so, this could reconcile the negative association with ever-use observed here alongside the positive association with current use reported by Kala et al. (7) and with Nyarko's (35) finding of greater contraceptive autonomy among wealthier Ghanaian women; however, this reconciliation is offered as a hypothesis and would require dedicated qualitative or longitudinal data to test.

The AUC advantage of Random Forest (0.865) over logistic regression (0.727) and LASSO (0.723) was statistically significant under a conservative comparison (z > 4.4, *p* < 0.001 for both), a 13.8-percentage-point improvement in discrimination that is likely to be programmatically meaningful for identifying women at low probability of prior contraceptive use. This advantage is attributable to Random Forest's capacity to model non-linear relationships, particularly for attitude and knowledge, and to detect interaction effects that logistic regression and LASSO cannot represent without explicit specification. The near-identical AUCs of logistic regression and LASSO indicate that, in this analysis, LASSO's principal contribution is variable selection and interpretability rather than improved predictive performance. Its elimination of age group, cultural barriers, and health worker attitude problems offers a cross-validated basis for simplifying future predictive models in comparable populations, and the retention of knowledge with a near-zero coefficient is consistent with its non-significant logistic regression *p*-value, even as Random Forest's importance ranking points to a meaningful, non-linear contribution.

Taken together, these results support a methodological recommendation for contraceptive behaviour research in low- and middle-income settings. Logistic regression remains necessary for adjusted, directly interpretable association estimates, but is usefully complemented by Random Forest, which can flag candidate non-linear or interaction effects that regression-based approaches may miss and that can subsequently be tested with explicitly specified interaction terms.

### Limitations

Several limitations should be considered when interpreting these findings. The cross-sectional design precludes causal inference; observed associations may reflect reverse causality (for example, positive attitudes toward contraception may follow from prior use rather than precede it) or residual confounding by unmeasured variables, including male partner characteristics, household-level religious practice, and community social norms. The facility-based sampling frame, while necessary for feasible recruitment, excludes women who do not access health facilities, a group that may differ systematically in contraceptive behaviour and attitudes from facility attendees; this limits generalisability to non-facility-attending women and to other sub-regions of Northern Ghana with different facility access profiles. The ever-use outcome captures lifetime initiation but cannot distinguish current users, past users who discontinued, and women who tried and abandoned contraception. This distinction is relevant to intervention targeting that a longitudinal design would be needed to resolve.

The knowledge scale's low internal consistency (*α* = 0.31) limits its interpretation as a unidimensional construct; while this is expected for a formative index spanning distinct knowledge domains (19), it also means that the composite score may combine items with heterogeneous, and possibly offsetting, associations with ever-use, which could partly explain its non-significance in the additive logistic model despite its high Random Forest importance. The analytical sample of 387 from 414 (93.5% retention) reduces but does not eliminate the risk of selection bias from listwise deletion; a formal comparison of sociodemographic characteristics between included and excluded participants was not conducted in this analysis and would strengthen future work using this dataset. Model evaluation in this study relied on discrimination (AUC). However, calibration, which assesses whether predicted probabilities correspond to observed outcome frequencies, was not assessed and should be examined before any of these models are used for programme-level risk targeting. Finally, Random Forest variable importance reflects predictive contribution rather than a causally interpretable, confounder-adjusted effect, and should be read accordingly throughout this paper.

## Conclusions

This study provides a three-model comparison of logistic regression, LASSO, and Random Forest for predicting contraceptive ever-use among women in Northern Ghana. Positive attitude toward contraception was the most consistently important predictor across all three approaches. Formal education, parity, and health worker information access were additional enabling factors identified by both regression-based models. Random Forest achieved better discrimination than either regression approach and highlighted knowledge score and age group as predictors whose contribution to classification was not evident from their logistic regression coefficients, a pattern consistent with non-linear or interaction-mediated association that warrants direct testing in future analyses. The inverse association between wealth and ever-use, which contrasts with the positive association reported for current use in this same population (7), illustrates that the causal dynamics of contraceptive initiation and continuation may differ meaningfully. The explanations proposed here, involving polygyny, religious affiliation, and partner dynamics, are hypotheses for future qualitative investigation rather than conclusions supported directly by the present data.

Programmatic implications include prioritising attitude-transformation strategies, such as peer counselling and structured myth-busting dialogue, alongside rather than in place of knowledge-based education; integrating contraceptive counselling into post-natal care platforms to capitalise on the parity-associated motivation window; and strengthening the content and quality of health worker counselling, given that health workers are already the dominant information source in this population. Machine learning methods such as Random Forest can usefully complement, though not replace, conventional regression in family planning research, provided their outputs are interpreted as predictive rather than causal evidence and are followed, where feasible, by calibration assessment and external validation.

## Data Availability

The raw data supporting the conclusions of this article will be made available by the authors, without undue reservation.
